# Stiffness and stability of bamboo stem- A optimal design perspective

**DOI:** 10.1016/j.heliyon.2024.e35403

**Published:** 2024-08-08

**Authors:** Mannan Sayyad, Bhanudas Bachchhav, Sachin Salunkhe, Lenka Cepova, Jiri Struz, Emad Abouel Nasr, Khaled M.S. Gad El Mola

**Affiliations:** a Department of Mechanical Engineering, AISSMS College of Engineering, Pune, India; b Department of Biosciences, Saveetha School of Engineering, Saveetha Institute of Medical and Technical Sciences, Chennai, India; c Gazi University Faculty of Engineering, Department of Mechanical Engineering, Maltepe, ANKARA, Turkey; d Department of Machining, Assembly and Engineering Metrology, Faculty of Mechanical Engineering, VSB—Technical University of Ostrava, 70800 Ostrava, Czech Republic; e Faculty of Mechanical Engineering, Department of Machine Parts and Mechanism, VSB – Technical University of Ostrava, 17. Listopadu 15/2172, CZ 708 33 Ostrava, Czech Republic; f Department of Industrial Engineering, College of Engineering, King Saud University, P.O. Box 800, Riyadh 11421, Saudi Arabia; g Department of Mechanical Engineering, Industrial Engineering Program, Helwan University, Helwan, Cairo, Egypt

**Keywords:** Optimal design, Bamboo, Functionally graded material, Mechanical integrity, Delamination, Stelar arrangement

## Abstract

During the evolution process, a bamboo stem achieves a significant height (up to 20 m) to fulfil its phototropic requirements. While on land, the stem is mostly subjected to bending load which makes it liable to fail by uprooting. However, this failure is prohibited by smart structure of bamboo stem which includes graded arrangement of fibre bundles in the cross-section and a tapered cantilever form of the stem. This paper attempts to understand the optimal design of bamboo stem through the relationship between the stellar arrangement of stiff fibre bundles in the cross-section and the tapered form. In this work, a comparison between two types of stellar arrangement, namely uniform and graded, is presented in view of non-linear bending analysis through elastica theory and fracture-induced delamination, both numerically. It is observed from the results that a bamboo stem prefers to evolve with graded stellar arrangement which provides gradation of stiffness and toughness over the cross-section; the trend in toughness being opposite to that of stiffness. Moreover, interplay of stellar arrangement and gradation of stiffness-toughness thereof is found to be the governing mechanism for ensuring its mechanical integrity and stability in view of an optimal design perspective. The smart structure of bamboo is recommended for bio-mimicking.

## 1 Introduction

The bamboo stem belongs to the Monocotyledon family of plants, which does not increase in size by forming cylindrical or annular rings. Such plants grow instead in a vertical direction with a tall, slender stem having a hollow circular cross-section tapered gently along the length. During the evolution process, the bamboo stem achieves a significant height (up to 20 m) to fulfil the phototropic requirements. It is not uncommon for such vertical structural members to bend under the action of wind load and buckle due to self-weight and large slenderness ratio. The bending of the stem is also associated with large deformations and rotations [1]. Thus, the stems are prone to critical failures affecting their mechanical integrity, stability and survival. The stems are supposed to carry out the mechanical function of supporting the soft tissues like water-carrying tubes and food storage cells [2].

As mentioned earlier, the stems in Monocotyledon plants are typically hollow and circular in cross-section. Structurally, the stems are idealised to thin-walled tubes, which are observed to be efficient members in bending due to arbitrary transverse loads. Moreover, the shape of the cross-section maximises flexural rigidity and minimises bending stresses [3]. By maximising flexural rigidity, the stem minimises the curvature along the length, an essential factor controlling stability [4]. In a separate study, Sivanagendra and Ananthasuresh [5] have discussed the optimisation of a dwarf wheat plant as a cantilever beam under deformation-dependent loads because of the resistance to uprooting and found that the plant to be compliant decreases Young's modulus selectively. Sivanagendra and Ananthasuresh [5] have analysed this by considering the large deflections with the help of co-rotational finite element analysis.

**Fig. 1:**
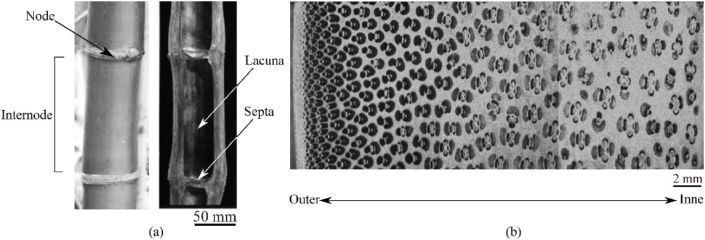
A bamboo shoot has been seen in the longitudinal section (a) and its transverse section (b), showing the peripheral stelar arrangement of fibre bundles.

Apart from the geometrical features, the overall behaviour of bamboo stems is also influenced by their anisotropic nature, as most biomaterials possess anisotropic elastic properties. Bamboo is radially graded transversely isotropic material. The near-crystalline cellulose molecules form long spiral chains almost aligned with the axial direction [6,7]. These chains, also called microfibrils, are the significant contributors to bulk material stiffness, which eventually evolves into dense, closely packed fibre bundles (see Fig. 1).

The arrangement of fibre bundles on the cross-section is the archetypal stellar arrangement. It can be seen from the micrographs of the cross-section that the fibre bundles are not distributed uniformly but have a smooth gradation, with more fibre bundles in the outer region. Sayyad et al. [8] have shown that the fibre density gradation strongly correlates with the graded axial stiffness. Thus, the bamboo stem is structurally a functionally graded material (FGM). Due to their enhanced properties, FGMs have been widely used in various engineering applications, such as marine and aerospace engineering. Many FGMs have been fabricated in the shapes of beams, and an elasticity solution has been sought for the deformation behaviour of FGM beams [9]. As far as bamboo stems are concerned, it is required to have a great extent of flexibility to accommodate the large deformation.

Obataya et al. [10] have discussed this aspect with the support of experimental results on split bamboo shoots and shown that the combination of the fibre-rich outer part and the compressible inner part was responsible for the flexural ductility. Several technical papers published so far discuss the bending characteristics of cantilever beams subjected to transverse loads. However, these studies need to be more comprehensive to highlight the significance of the non-uniform arrangement of fibre bundles in the bamboo stem. In this article, large deflections of a flexible tapered cantilever beam subjected to a tipping load are studied with two archetypal stelar arrangements in comparison, with uniform distribution and graded distribution. The analysis is carried out within the framework of the nonlinear deformation of a tapered elastic cantilever due to a tip load. The exact shape of the deflection curve of a flexible member is called the elastica.

Hu et al. [11] have discussed super-strong biomimetic bulk bamboo-based composites by a neural network interfacial design strategy. Herein, a neural network interface design strategy has been proposed, and a mechanical dissociation and partial matrix removal pre-treatment method has been used to open the weak intercellular layer and bamboo cell wall layer to increase the resin permeation channels. This has allowed the resin to form a multi-scale bonding interface between multiple dense bamboo layers, achieving the preparation of bulk bamboo-based composite with adjustable dimensions and properties.

In another study, Xia et al. [12] have prepared multi-layered microcapsule-shaped activated biomass carbon from bamboo parenchyma cells for energy storage and cationic dyes removal. This enables the biomass material to have an ultrahigh surface area (3973 m^2^g^−1^) and pore volume (2.076 cm^3^g^−1^) after activation. Moreover, the biomass carbon has been shown a maximum adsorption capacity of 1750 mgg^−1^ in methylene blue dye and demonstrates excellent continuous adsorption performance of continuous flow method. These results confirm that the biomass carbon from bamboo parenchyma cells has great potential for energy storage and cationic dyes removal.

ISO 22156 applies to the load bearing structure made of round bamboo or shear panel systems in which the framing members are made from round bamboo. This document is concerned only with requirements for mechanical resistance, serviceability and durability of bamboo structures. On the other hand, ISO 22157 specifies test procedures for specimens obtained from round bamboo culms. Both of these standards are intended for bamboo structures, design, and properties. Since, the objective of the present work is to understand and establish the correlations between the anatomical structure and the mechanical integrity of bamboo stem, these standards are not referred in the work.

In the present paper, Sec. 2 presents a discussion of the nonlinear deformation of the elastic cantilever concerning the static analysis. The deflection analysis is performed for two configurations corresponding to homogenous and graded stelar arrangement. The transversely isotropic homogeneous and radially graded properties are used to perform simulations on a 10 m long tapered cantilever subjected to a tip load in Sec. 3. These simulations provide the deflection and stress profiles while undergoing large-scale bending. A plausible reason for the graded stelar arrangement of fibre bundles in bamboo is discussed in Sec. 4. The conclusions from the work are reported in Sec. 5.

## 2 Nonlinear deformation of a tapered elastic cantilever

### 2.1 The elastica theory

The design of aircraft or aerospace structures is primarily controlled by minimum weight criterion applied to various polymeric materials subjected to static and dynamic loads. Being flexible, these structures undergo large deformations which are highly nonlinear and are complex to achieve the solution within the framework of linear elasticity. The solution becomes further complicated when the structural components have variable cross-sectional dimensions and elastic properties. For example, bamboo stem, widely used as a structural material in scaffoldings, has variable cross-sectional dimensions and elastic properties graded along the radial direction. When subjected to heavy wind loads, the stems prefer to undergo large deflections to reduce the moment Armand, hence lowering the uprooting moment, thereby increasing the chances of survival.

**Fig. 2:**
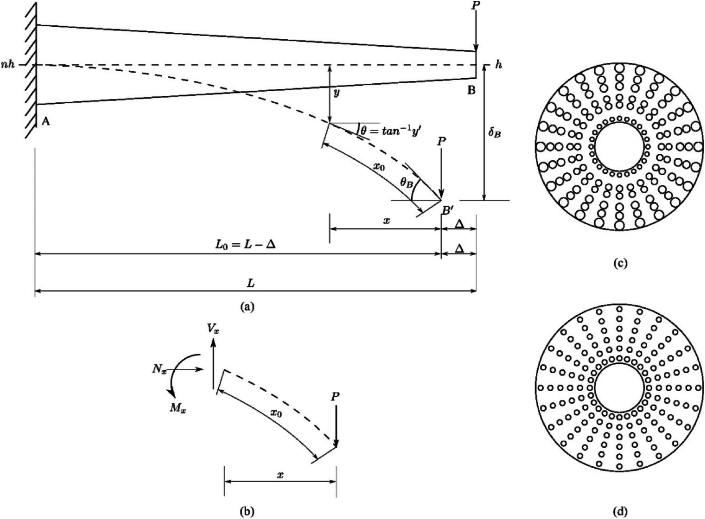
(a) A tapered cantilever beam with a point load P at the free end. (b) An elemental free-body diagram of the beam. Commonly observed stellar arrangements. (c) Gradation in stelar arrangement of bundles and (d) Uniformly distributed bundles.(1)$\frac{1}{R}=\frac{M}{EI}$

Consider an elastic cantilever beam subjected to a tip load P, as shown in Fig. 2(a) and a small part of the length in a deformed configuration ($x_{0}$) with its free body diagram, as shown in Fig. 2(b). $x_{0}$ is given by $x_{0}=\int_{0}^{x}{\left\{1+{\left[\frac{dy}{dx}\right]}^{2}\right\}}^{1/2}dx$. Due to nonlinear bending, there is a difference between the projected length x and the length $x_{0}$ along the deformed beam. According to Euler-Bernoulli theory, the bending moment M is varies with the curvature 1/R, which can be expressed aswhere R is the radius of curvature, E is the modulus of elasticity, and I is the cross-sectional moment of inertia [1]. Eq. (1) can also be written in a Cartesian coordinate system as

(2) $$\frac{1}{R}=\frac{\left(d^{2}y/dx^{2}\right)}{{\left[1+{\left(\frac{dy}{dx}\right)}^{2}\right]}^{3/2}}=\frac{M}{EI}\text{,}$$

where y is the deflection defined as y=y(x). Within the linear elasticity framework, ${\left[1+{\left(\frac{dy}{dx}\right)}^{2}\right]}^{3/2}\approx 1$ and an exact solution can be easily sought. However, for the large deflections in flexible beams discussed earlier, Eq. (2) is nonlinear, and it is not easy to obtain the exact solution [1]. The task becomes more complex for variable geometry and elastic properties, i.e. E=E(x)andI=I(x). Under these conditions, the Euler-Bernoulli equation is written as

(3) $$\frac{\left(d^{2}y/dx^{2}\right)}{{\left[1+{\left(\frac{dy}{dx}\right)}^{2}\right]}^{3/2}}=\frac{M_{x}}{E_{x}I_{x}}\text{.}$$

For the arbitrary loading, $E_{x}\;and\;I_{x}$ can vary, and without losing generality, Eq. (3) can be re-written as

(4) $$\frac{\left(d^{2}y/dx^{2}\right)}{{\left[1+{\left(\frac{dy}{dx}\right)}^{2}\right]}^{3/2}}=\frac{M_{x}}{E_{0}f\left(x\right)I_{0}g\left(x\right)}\text{,}$$

where $E_{0\;}\text{and}\;I_{0}$ are reference values and f (x)=g(x)=1, if EandI are constant. The term representing the curvature in Eq. (1) depends on the deformed shape of the beam, and it is required to compute the quantity on the right-hand side also in the deformed configuration. The transverse displacement can be written by integrating Eq. (4) twice as

(5) $$y\left(x\right)=\frac{1}{E_{0}I_{0}}\int \left\{-\int {\left[1+{\left(\frac{dy}{dx}\right)}^{2}\right]}^{3/2}\frac{M_{x}}{f\left(x\right)g\left(x\right)}\right\}dx+C_{1}\int dx+C_{2}\text{,}$$

where $C_{1}$ and $C_{2}$ can be conveniently obtained knowing the boundary conditions. This system can be expressed as an equivalent linear and pseudo-linear system to get the exact solution to Eq. (5). To achieve this, a beam of constant rigidity $E_{0}I_{0\;}$ is considered with an identical length to the one described in Fig. 2(a). A similar expression for transverse displacement of the equivalent system ($y_{e}$) can be written as

$$y_{e}\left(x\right)=\frac{1}{E_{0}I_{0}}\int \left\{-\int {\left[1+{\left(\frac{dy_{e}}{dx}\right)}^{2}\right]}^{3/2}\frac{M_{x}}{f\left(x\right)g\left(x\right)}\right\}dx+C_{1}^{\prime }\int dx+C_{2}^{\prime }\text{.}$$

Obviously, the deflections obtained as y and ye will be identical provided $C_{1=}C_{1}^{\prime }$ and $C_{2=}C_{2}^{\prime }$. With this, the identity will be satisfied for

(6) $$M_{e}=\frac{M_{x}}{f\left(x\right)g\left(x\right)}\text{,}$$

or more specifically

(7) $${\left[1+{\left(\frac{dy_{e}}{dx}\right)}^{2}\right]}^{3/2}M_{e}={\left[1+{\left(\frac{dy}{dx}\right)}^{2}\right]}^{3/2}\frac{M_{x}}{f\left(x\right)g\left(x\right)}\text{.}$$

By solving Eq. (3) for $d^{2}y/dx^{2}$, we obtain

(8) $$\frac{d^{2}y}{dx^{2}}=\frac{1}{E_{0}I_{0}}{\left[1+{\left(\frac{dy}{dx}\right)}^{2}\right]}^{3/2}\frac{M_{x}}{f\left(x\right)g\left(x\right)}\text{.}$$

By substituting Eq. (6) and Eq. (7) in Eq. (8), we get

(9) $$\frac{d^{2}y}{dx^{2}}=\frac{M_{e}^{\prime }}{E_{0}I_{0}}\text{.}$$

Equation (9) represents the governing equation of a pseudo-linear equivalent system. Once the new system's bending moment diagram $M_{e}^{\prime }$ is completely known, the equation can be solved as a linear differential equation. The deflections obtained by solving pseudo-linear systems are identical to the original nonlinear system.

### 2.2 Variable stiffness tapered cantilever beam with a point load at the free end

The reference values of the moment of inertia $I_{0}$ and Young's modulus $E_{0}$ may be selected at the beam's free end. Due to the horizontal displacement of the free end Δ, the moment of inertia I(x) for 0≤x≤(L−Δ) is written as

(10) $$I\left(x\right)=I_{0}{\left[1+\left(n-1\right)\frac{x}{L-\Delta }\right]}^{3}=I_{0}g\left(x\right)\text{,}$$

where n is the taper constant. By substituting Eq. (10) into Eq. (1) we get

(11) $$\frac{\left(\frac{d^{2}y}{dx^{2}}\right)}{{\left[1+{\left(\frac{dy}{dx}\right)}^{2}\right]}^{3/2}}=\frac{1}{E_{0}I_{0}}\frac{M_{x}}{f\left(x\right){\left[1+\frac{\left(n-1\right)}{L}\int_{0}^{x}{\left\{1+{\left(\frac{dy}{dx}\right)}^{2}\right\}}^{1/2}dx\right]}^{3}}\text{,}$$

or

(12) $$\frac{\left(\frac{d^{2}y}{dx^{2}}\right)}{{\left[1+{\left(\frac{dy}{dx}\right)}^{2}\right]}^{3/2}}=\frac{{\left(L-\Delta \right)}^{3}}{E_{0}I_{0}}\frac{M_{x}}{f\left(x\right){\left[1+\left(n-1\right)\frac{x}{L-\Delta }\right]}^{3}}\text{,}$$

Equation Eq. (12) is a nonlinear differential equation simplified for a flexible cantilever beam and easier compared to Eq. (11) to obtain accurate results.

## 3 Uniform and variable stiffness tapered cantilever flexible beam

In this section, two flexible tapered cantilever beams made of uniform and variable stiffness are compared. The motivation of this study is to understand the mechanical advantage bamboo stem obtained from the graded stelar arrangement (see Fig. 1(b)). To achieve this, two stelar arrangements with ‵vascular bundles', as shown in Fig. 2(c) and (d), are considered. It can be observed that the arrangement in Fig. 2(d) is advantageous from the axial stiffness point of view, as the vascular bundles are of equal size and are uniformly distributed. Moreover, it results in a uniform distribution of self-weight to avoid so-called Euler buckling [4].

The load at which the buckling occurs is proportional to rigidity $E_{L}I$, where $E_{L}$ is axial Young's modulus and I are the moment of inertia. For the arrangement shown in Fig. Fig. 2(c), Young's modulus *E* is graded and weak in buckling. However, the bamboo stem has periodic partitions placed along the length (see Fig. 1(a)) that prevent Euler buckling.

**Fig. 3:**
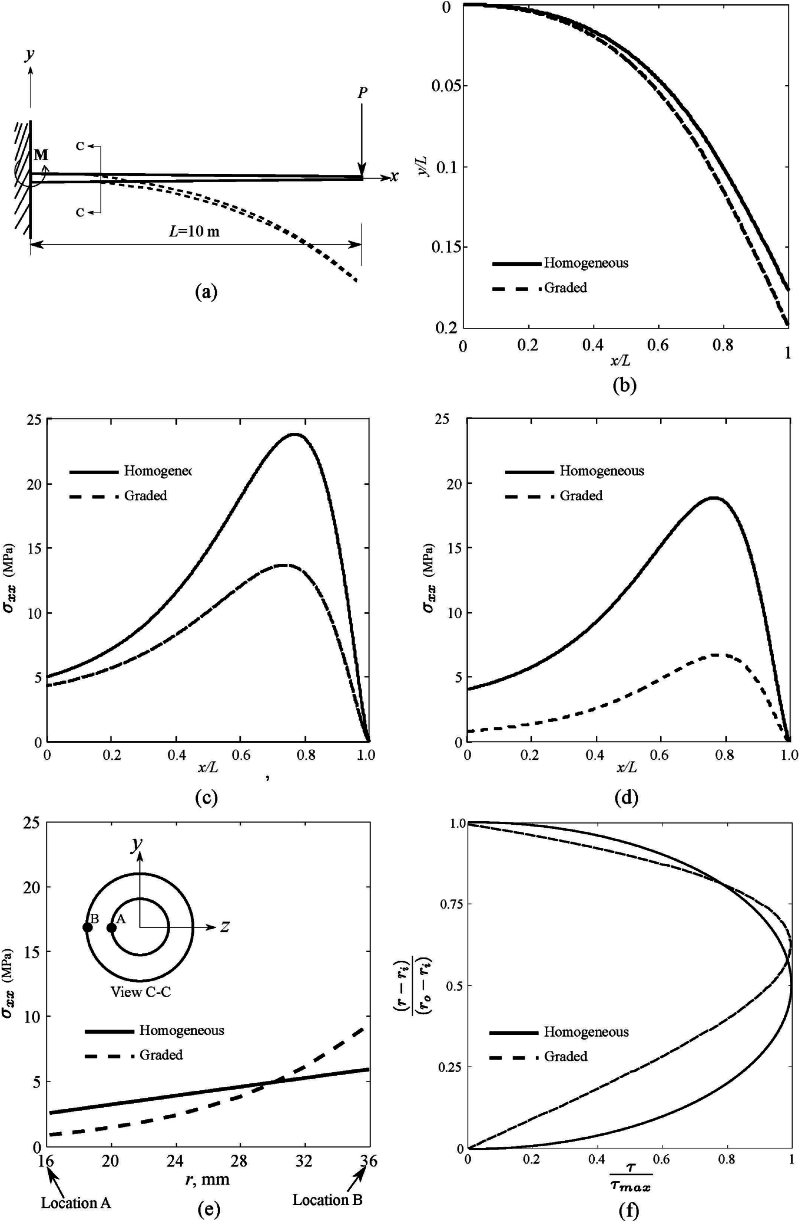
(a) The structural model of a tapered beam with tip load. Variations of deflection with beam length are shown in (b). Axial stress (*σ*_xx_) variation along the outer surface length is plotted in (c). Similarly, the interpretation of axial stress (*σ*_xx_) on the inner surface is plotted in (d). The distribution of shear stress (*τ*) and longitudinal stress (*σ*_xx_) over the thickness at x = 0 are plotted in (e) and (f).

The advantages of the stellar arrangement as shown in Fig. 1(b) need to be clarified. To highlight this, it is worthwhile to compare and discuss uniform and variable stiffness tapered cantilever flexible beams subjected to bending, as shown in Fig. 3(a). The values of elastic properties are given in Table 1. The structural models for cantilever beams are simulated for P=100N following the procedure discussed in Sec. 2. Here, two cases are analysed:

i. A 10 m long tapered cantilever beam with variable properties in the cross-section, a ‘graded’ case.
ii. An equally long cantilever with properties equal to the average over the cross-section. This is a ‘homogeneous’ case.

**Table 1:** Elastic properties of two materials for Graded and Homogeneous cases. The data are obtained by the method outlined in Sayyad et al. [8].

		‘Graded’ case	‘Homogeneous’ case
Young's modulus (GPa)	Axial	$1.23e^{0.08r}$	10.9
	Transverse	$0.56e^{0.016r}$	0.85
Shear modulus (GPa)	Axial	$0.5e^{0.05r}$	1.9
	Transverse	$0.46e^{0.03r}$	1.02
Poisson's ratio	Axial	0.36	
	Transverse	0.19	

A suitable form of variation of the elastic properties, ∼A exp (kr), is adopted all along the length of the bamboo. The structural models for bamboo are shown in Fig. 3 (a). These models are simulated in ABAQUS/Standard using 8-node linear brick elements and the user material described earlier. The cantilever beams are subjected to the wind load given by:

$$v=v_{0}{\left(\frac{x}{x_{0}}\right)}^{\frac{1}{14}}$$

In the present work, realistic parameters were chosen for $v_{0}$ and $X_{0}$ as 16 m/s and 10 m, respectively [13]. First, the deformed shapes of the beams are studied and plotted, as shown in Fig. 3(b). These results clearly indicate the reason for bamboo shoots being tapered.

It can be seen that the deflection of the graded beam has increased by 12.5 %, which is associated with the increased deflection in x direction, as the beam cannot change the length. While achieving the more significant deflection, the graded beam reduces the moment at the support. For the ‘homogeneous’ case, the moment at the root is larger than the ‘graded’ case. This shows that, a tall homogeneous structure is most likely to be failed by wind loads [3]. This mechanism is also observed in many other plants, Metridium (see, pp 376, [13]).

To gain further insights, looking at the axial stress in beam bending is worthwhile. For this, axial and shear stress are compared, and the stress distribution is plotted in Fig. 3(c)–(f). It can be seen from Fig. 3(c) that axial stress $\sigma_{xx}$ on the outer surface increases monotonically and attains a peak value at about x/L=0.8 for both 'homogeneous' and 'graded' cases, the stress being on the lower side, approximately half, in the 'graded' case. On the inner surface as seen in Fig. 3(d), $\sigma_{xx}$ has a similar trend, but the stress level is reduced by approximately 75 % in the 'graded' case. The gradation of properties has a twofold advantage: considerably reducing bending stress levels.

Bamboo has a soft core made of cellular parenchymatous material, which is weaker than stiff fibre bundles (see Fig. 1). Thus, the graded stelar arrangement seems to elevate the bending stress at the outer surface, which is dominated by stiff fibre bundles capable of withstanding the stress. On the other hand, the mechanism protects weaker parenchymatous cells. These cells are in the form of a closed-cell foam structure, which can absorb large compressive deformations.

Thus, by placing the stiff fibre bundles in the periphery (see Fig. 2(c)), large deformations are allowed along with the redistribution of the bending stress over the cross-section. The fact is validated by the distribution of axial stress ($\sigma_{xx}$) over the thickness, as shown in Fig. 3(e). It can be observed that for both 'homogeneous' and 'graded' beams, the axial stress is monotonically varying from the inner region (point 'A' to 'B'), and the average value of the stress might be the same in both cases. There is stress redistribution over the cross-section by which stress is lowered in mechanically weak regions, elevating it to stiff regions.

This phenomenon protects the bamboo stem from premature failure. The axial stresses are the highest at around 4/5th height of the stem, so when a bamboo stem breaks, it will fail in this region. However, from the deformation profile shown in Fig. 3(b), it is evident that for x/L=0.8, the beam is almost aligned with the direction of the applied load. This might reduce the effective load on the stem that causes bending. Moreover, the other component of the load acts as a shear load on the surface and has less potential to cause damage. Hence, the chances of stem failure are lowered.

According to Euler-Bernoulli's beam theory, the shear stress is maximum at the neutral axis. The shear stress distribution over the stem thickness is plotted in Fig. 3(f) for completeness. It is clear that for the ‘homogeneous’ case, the location of the neutral axis (corresponding to maximum shear stress) is at the middle of the section. In contrast, it is shifted towards the outer periphery, where fibre bundles are concentrated. This results in protecting the stem section from shear failure under bending.

## 4 Stability of bamboo

In the previous sections, it has been pointed out that the gradation in the stellar arrangement of fibre bundles is advantageous from a strength and stiffness point of view. When bamboo stems are subjected to heavy wind loads, they tend to deform non-linearly and undergo large deflections instead of getting uprooted. The bending stress usually initiates the surface cracks, which, when propagated, lead to catastrophic failure of heavy structures. The failure is typically a fracture of the structure due to incipient cracks. Tall plants like bamboo are also prone to this kind of failure. However, as far as bamboo is considered, the stem has periodic rings along the length to arrest the cracks. It is interesting to understand the role of gradation of mechanical properties in the stability of bamboo stems. Bamboo has a typical asymmetric gradation of stiffness and toughness, the opposite trends [3].

**Fig. 4:**
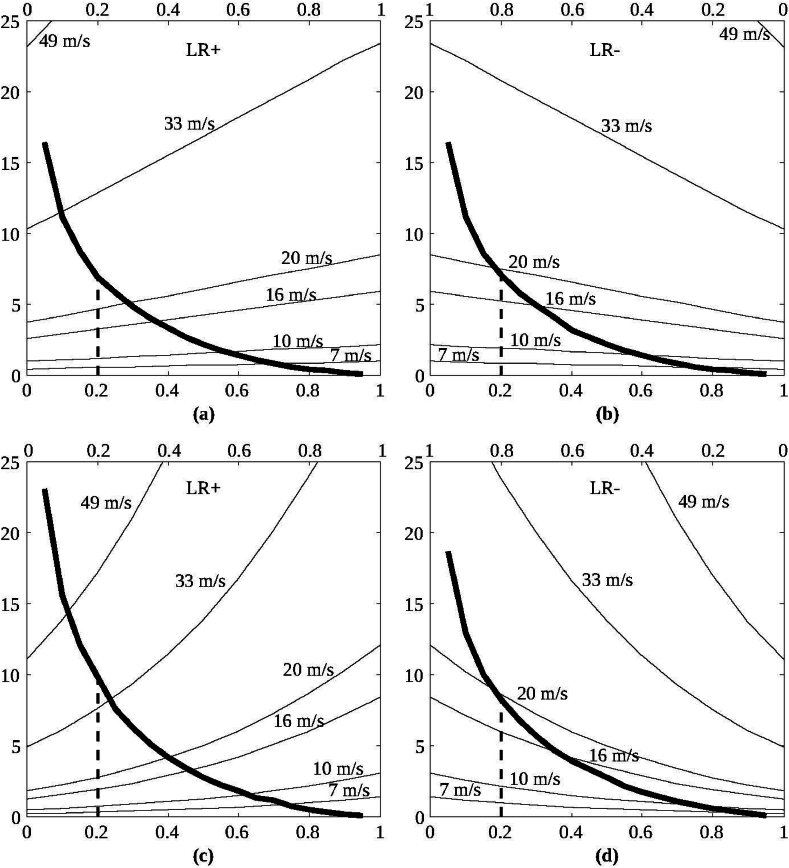
Comparisons of far-field stresses required to originate inter-laminar crack. (a) Surface crack from the inner surface in a ‵homogeneous' beam and (b) from the outer surface. The same cracks in the ‵graded' beam are shown in (c) and (d). On the Y-axis, stress is plotted, while the lower and upper X-axes represent the section's normalised crack width and normalised thickness, respectively.

Sayyad et al. [3] have characterised the fracture toughness of bamboo in various crack propagation modes and found that the toughness is also graded over the cross-section of the stem. The observations reported there are relevant to the present study, which is discussed here. Fig. 4 shows the variation of remote stress at the incipient crack required to propagate the crack and eventually leads to the failure of the structure. The axial stress distribution for various wind velocities is over-plotted for better understanding. A comparison is made between 'homogeneous' and 'graded' beams subjected to wind load.

As far as the present work is concerned, the asymmetric distribution of stiffness and toughness is well correlated with the stability of flexible tapered structures like bamboo stems. In light of the discussion, Fig. 4 leads to the plausible answer to the radially graded properties of bamboo stems. It can be seen from Fig. 4(a)–(b) that, if bamboo cross sections possess uniformly distributed fibre bundles, the surface crack will transform into an inter-laminar crack when the wind speed is approximately 18 m/s, whereas in the graded case, the wind speed level increases to about 41 m/s, as seen from Fig. 4(c)–(d). For the smaller crack sizes, the magnitude of wind speeds is even larger for the failure to happen.

It has been concluded that a crack originating from the inner surface is approximately 5 times tougher than the one from the outer surface. The toughness is much higher than the interlaminar fracture toughness [14]. Thus, a surface crack will convert into an inter-laminar crack which is further controlled by the relative difference in magnitudes of the toughnesses. If a surface crack propagates through the thickness of the stem, it will break the stem. However, by transforming to an inter-laminar crack, only a small part of the cross-section is lost instead of a complete failure, thereby reducing the extent of the failure in bending.

## 5 Conclusions

Bamboo stems are graded materials which is attributed to the stelar arrangement of fibre bundles, fibre bundles being dense at the outer periphery. The elastic properties are also graded accordingly which makes the material ‵radially graded transversely isotropic'. Moreover, inner surface is tougher than the outer. It is exactly opposite to the trend of variation of moduli.

The Elastica performance of tapered flexible cantilever beams is studied and compared for deflection and flexural stress. It is shown that the 'graded' arrangement outperforms the ‵homogenous' one in bending. The tall plants are prone to failure initiated by large deflections in bending. In the 'graded' case, the bending stress is redistributed such that weaker parenchymatous cells are protected from damage with stiff fibre bundles in the outer periphery bearing the bending stress.

Thus, the typical stelar arrangement of fibre bundles over the radius in bamboo stems has two purposes. The chances of uprooting the structure are lower in the ‘graded’ case than in the ‵homogenous' case. Moreover, under bending loads, the surface cracks, however very small, propagate as inter-laminar cracks along the axial direction, thereby prohibiting the failure of the entire stem with a small loss in flexural stiffness.

The interplay of stelar arrangement, tapering of diameter and asymmetric distribution of stiffness and toughness result in superior strength and enhanced bamboo stability, proving to be an efficient structural biomaterial.

The hypotheses presented in the work and its validation through numerical simulations is useful in mimicking of bamboo culms for structural design. Functionally graded material is one such material inspired from bamboo microstructure. Development of high-performance bamboo steel derived from natural bamboo is one sophisticated example in this regard.

## Funding statement

The authors extend their appreciation to 10.13039/501100002383King Saud University for funding this work through Researchers Supporting Project number (RSP2024R164), 10.13039/501100002383King Saud University, Riyadh, Saudi Arabia. This article was co-funded by the European Union under the REFRESH – Research Excellence For REgion Sustainability and High-tech Industries project number CZ.10.03.01/00/22_003/0000048 via the Operational Programme Just Transition and has been done in connection with project Students Grant Competition SP2024/087 “Specific Research of Sustainable Manufacturing Technologies” financed by the 10.13039/501100001823Ministry of Education, Youth and Sports and Faculty of Mechanical Engineering VŠB-TUO. Article has been done in connection with project Students Grant Competition SP2024/087 “Specific Research of Sustainable Manufacturing Technologies” financed by the Ministry of Education, Youth and Sports and Faculty of Mechanical Engineering VŠB-TUO.

## Data availability statement

No data was used for the research described in the article.

## CRediT authorship contribution statement

**Mannan Sayyad:** Writing – original draft, Validation, Supervision, Software, Project administration, Methodology, Data curation, Conceptualization. **Bhanudas Bachchhav:** Writing – original draft, Supervision, Software, Resources, Project administration, Investigation, Formal analysis, Data curation. **Sachin Salunkhe:** Writing – review & editing, Writing – original draft, Validation, Resources, Project administration, Investigation, Funding acquisition. **Lenka Cepova:** Writing – review & editing, Writing – original draft, Validation, Resources, Project administration, Methodology, Data curation. **Jiri Struz:** Software, Resources, Methodology, Investigation, Funding acquisition, Formal analysis, Data curation. **Emad Abouel Nasr:** Writing – review & editing, Validation, Supervision, Resources, Project administration, Funding acquisition. **Khaled M.S. Gad El Mola:** Writing – original draft, Software, Resources, Project administration, Investigation, Formal analysis.

## Declaration of competing interest

On behalf of all authors, the corresponding author states that there is no conflict of interest.
