# Corrigendum to “Stiffness and stability of bamboo stem- A optimal design perspective” [Heliyon Volume 10, Issue 16, August 2024, Article e35403]

**DOI:** 10.1016/j.heliyon.2025.e43273

**Published:** 2025-04-01

**Authors:** Mannan Sayyad, Bhanudas Bachchhav, Sachin Salunkhe, Lenka Cepova, Jiri Struz, Emad Abouel Nasr, Khaled M.S. Gad El Mola

**Affiliations:** a Department of Mechanical Engineering, AISSMS College of Engineering, Pune, India; b Department of Biosciences, Saveetha School of Engineering, Saveetha Institute of Medical and Technical Sciences, Chennai, India; c Gazi University Faculty of Engineering, Department of Mechanical Engineering, Maltepe, Ankara, Turkey; d Department of Machining, Assembly and Engineering Metrology, Faculty of Mechanical Engineering, VSB—Technical University of Ostrava, 70800, Ostrava, Czech Republic; e Faculty of Mechanical Engineering, Department of Machine Parts and Mechanism, VSB – Technical University of Ostrava, 17. Listopadu 15/2172, CZ 708 33, Ostrava, Czech Republic; f Department of Industrial Engineering, College of Engineering, King Saud University, P.O. Box 800, Riyadh, 11421, Saudi Arabia; g Department of Mechanical Engineering, Industrial Engineering Program, Helwan University, Helwan, Cairo, Egypt

In this article, Equation 5 contains an error:

Equation (5):

$$y\left(x\right)=\frac{1}{E_{0}I_{0}}\int \left\{-\int {\left[1+{\left(\frac{dy}{dx}\right)}^{2}\right]}^{3/2}\frac{M_{x}}{f\left(x\right)g\left(x\right)}\right\}dx+C_{1}\int dx+C_{2}\text{,}$$

$$y_{e}\left(x\right)=\frac{1}{E_{0}I_{0}}\int \left\{-\int {\left[1+{\left(\frac{dy_{e}}{dx}\right)}^{2}\right]}^{3/2}\frac{M_{x}}{f\left(x\right)g\left(x\right)}\right\}dx+C_{1}^{\prime }\int dx+C_{2}^{\prime }\text{.}$$

The correct version of Equation 5 should be as below:

(5) $$y\left(x\right)=\frac{1}{E_{0}I_{0}}\int \left\{-\int {\left[1+{\left(\frac{dy}{dx}\right)}^{2}\right]}^{3/2}\frac{M_{x}}{f\left(x\right)g\left(x\right)}dx\right\}dx+C_{1}\int dx+C_{2}\text{,}$$

$$y_{e}\left(x\right)=\frac{1}{E_{0}I_{0}}\int \left\{-\int {\left[1+{\left(\frac{dy_{e}}{dx}\right)}^{2}\right]}^{3/2}\frac{M_{x}}{f\left(x\right)g\left(x\right)}dx\right\}dx+C_{1}^{\prime }\int dx+C_{2}^{\prime }\text{.}$$

The authors apologize for the errors.

## Declaration of competing interest

On behalf of all authors, the corresponding author states that there is no conflict of interest.

